# A dynamic pandemic model evaluating reopening strategies amid COVID-19

**DOI:** 10.1371/journal.pone.0248302

**Published:** 2021-03-26

**Authors:** Ling Zhong

**Affiliations:** Department of Economics, Cheung Kong Graduate School of Business, Beijing, China; Univerza v Mariboru, SLOVENIA

## Abstract

Among over 200 COVID-19 affected countries, some are fighting to “flatten the curve”, while some others are considering reopening after lockdown. It remains unclear how different reopening strategies obstruct the local virus containment and impact the economy. We develop a model with travelers across heterogeneous epicenters. A low-risk area attempts to safely reopen utilizing internal policies, such as social distancing and contact tracing, and external policies, including capacity quota, quarantine, and tests. Simulations based on the COVID-19 scenario show that external policies differ in efficacy. They can substitute each other and complement internal policies. Simultaneous relaxation of both channels may lead to a new wave of COVID-19 and large economic costs. This work highlights the importance of quantitative assessment prior to implementing reopening strategies.

## Introduction

The global outbreak of COVID-19 leads to nation-wide lockdown in many countries that try to contain the virus spread. The countries and areas that have passed the worst phase of the outbreak or were able to prevent an internal outbreak are eager to reopen as prolonged lockdown could cause damages to the economy and social structure [1–3]. As a result, reopening strategies, especially their consequences on local people’s health, top the agenda of many local and national governments across the world, and have been discussed on social media among the general public. However, a pandemic model with multiple epicenters allowing for simulated outcomes to guide policy design in the process of reopening is lacking.

Existing literature prior to the COVID-19 outbreak has explored multiple-region epidemic models and analyzed past epidemics to understand large-scale pandemics and policy interventions in theory and practice. Theoretically, SIR or SEIR models with travelers across regions have been developed to describe epidemics with multiple epicenters without policy interventions [4–7]. These models showed the important roles played by travelers in spreading out of virus diseases, and the positive effect of travel bans on slowing down the spreading of infectious diseases across regions. A few papers focused on modeling the effects of physical distancing and contact tracing on containing epidemics [8], or the effect of travel bans on preventing an outbreak that affects many regions [9]. Practically, many papers have been published to investigate the outbreak and the containment of recent pandemics, such as SARS, the Ebola virus disease, the influenza A virus (H1N1), the Hong Kong influenza, and the seasonal flu in North America. Empirical analyses on SARS showed the effectiveness of quarantine and other internal policies [10–13]. Another study on SARS provided evidence for the good performance of combining isolating infected patients with screening travelers [14]. The studies on various influenza and Ebola empirically examined the contribution of travelers to pandemic outbreaks, and the effects of travel bans and screening for travelers on containing the virus [15–18]. Since the COVID-19 pandemic outbreak, several studies are particularly relevant to our work. Papers using data from the outbreak in Wuhan showed that both traveler flow across regions and human mobility within the city are strongly correlated with geographical distribution of new cases, and local control measures such as quarantine may disentangle this correlation [19, 20]. Another paper focusing on policies in Wuhan estimated the effect of physical distancing [21]. Using data beyond Wuhan, researchers showed that quarantine is more effective than travel bans in slowing down the virus from spreading to multiple regions [22], and that quarantine orders suppress travel demands, mitigating the outbreaks beyond the epicenters of the pandemic [23]. Lastly, in addition to public health researches, some papers study the economic consequences of epidemic outbreaks and containment policies [24]. As mentioned in the limitation of existing papers, e.g. [17], a general model allowing policy makers to illustrate the interplay between internal and external policies on containing the outbreak is lacking. There is also very little work on the reopening strategy for former epicenters, as most researchers focused on the outbreak progress and policies that stop the virus from spreading out. The model framework we propose fills in both gaps.

In this paper, expanding previous researches, we build a model for a realistic scenario in today’s context, in which an area with low domestic epidemic risk wants to reopen its border amid risks of importing cases from multiple areas. In the model setting, the virus spreading of each area depends on the nature of the virus, the area’s internal and external policies, and the characteristics of inbound travelers. This model setup highlights the impact of government initiative in the low risk area, allowing us to answer a set of very timely questions asked by academic researchers and policymakers: what are the quantitative effects of various reopening strategies? How do specific reopening policies interact? Does the public health objective always disagree with the economic objective? This paper contributes to the literature by bridging epidemiology and public policy to evaluate proposed government interventions and to provide quantitative insights for policy design amid any pandemic. It also contributes to the reopening strategy discussion amid the COVID-19 pandemic by demonstrating the functionality of the theoretical model with real-world data.

## Methods

In this paper, we develop a model framework incorporating multiple areas that are in different stages of the pandemic outbreak and are connected by travelers, and calibrate the model based on real-world data. The model features time-varying policies in an area with relatively lower pandemic risk. The policies include social distancing that affects infection rate, contact tracing ability that determines the contagious level of infected people, the accuracy of the tests, the number of travelers allowed to come in from each type of area, and the quarantine and testing policy on the travelers.

A list of notations used in the model is provided in Table 1. When we refer to parameters without time subscript in this paper, it means the parameter takes a constant value over time.

**Table 1:** Notations.

Notation	Description
*In the standard SIR model*	
*S*_*t*_	Number of susceptible people in period *t*
*I*_*t*_	Number of infected people in period *t*
*R*_t_	Number of recovered people in period *t*
*N*	Population size, i.e. *N* = *S*_*t*_+*I*_*t*_+*R*_*t*_ for all *t*
*β*	Transmission rate
*γ*	Recovery rate
*Disease parameters in the transition matrix ($P_{t}^{i}$) of the expanded model*	
*ρ*	Reinfection rate, i.e. the transmission rate for recovered people
*μ*	Diagnosis rate
*τ*	Severe symptom development rate
*δ*	Mortality rate of people with severe symptoms
*γ*_1_	Recovery rate for asymptomatic patients
*γ*_2_	Recovery rate for patients with mild symptoms
*γ*_3_	Improvement rate, the rate at which severe symptoms disappear
*Government’s internal policy parameters and functions*	
$g_{t}^{i}$	Social distancing parameter
$c_{t}^{i}$	Contact tracing and random testing parameter
$h_{t}^{i}$	Quarantine probability of asymptomatically infected people. It is a function of $c_{t}^{i},\;\theta_{t}^{i}$, and $g_{t}^{i}$.
$\sigma_{t}^{i}$	Self-quarantine exit rate. It is the rate at which susceptible people in self-quarantine rejoins the local community
${sq}_{t}^{i}$	Self-quarantine intensity parameter, reflecting the government policy on asking susceptible people to self-quarantine
$w_{t}^{i}$	Quarantine probability of susceptible people. It is a function of ${sq}_{t}^{i}$.
$\theta_{t}^{i}$	Effectiveness of one test in area *i* in period *t*, depending on technology advancements
*Travel related parameters and matrices in the expanded model*	
*N*_*i*_	Population size of area *i* in the initial state
$N_{t}^{ij}$	Number of travelers from area *i* to area *j* in period *t*
${travel}_{t}^{ij}$	Distribution across the 8 epidemic states of travelers from area *i* to area *j* in period *t*. Numbers of people in all states sum to $N_{t}^{ij}$.
${\hat{P}}_{t}^{i}$	Testing matrix of area *i* in period *t*, reflecting the test effectiveness $\theta_{t}^{i}$
$\tilde{P}$	Quarantine matrix of area *i*. The matrix only captures changes in traveler’s health status and does not involve local transmission.
$P_{t}^{ij}$	Local transmission matrix among travelers from area *i* to area *j* in period *t*
$q_{t}^{i}$	Quarantine duration for inbound travelers in area *i* who arrived in period *t*
$r_{t}^{i}$	Number of tests for inbound travelers in area *i* in period *t*

The virus transmission within each area follows an expanded version of the Susceptible–Infectious–Recovered (SIR) model, in which the infectious state of the standard SIR model is divided into asymptomatic and symptomatic. The expanded model allows some susceptible and asymptomatic infectious people to be quarantined. We show the steps of expanding the three-state standard SIR model to the eight-state model as follows. First, the discrete-time standard SIR model is given by Eqs (1) and (2) [25].

$$\left[\begin{array}{c} S_{t+1}\\ I_{t+1}\\ R_{t+1} \end{array}\right]=P_{t}^{SIR}\times \left[\begin{array}{c} S_{t}\\ I_{t}\\ R_{t} \end{array}\right]$$ (1)

in which the transition matrix is

$$P_{t}^{SIR}=\left[\begin{array}{ccc} 1-\frac{\beta I_{t}}{N} & 0 & 0\\ \frac{\beta I_{t}}{N} & 1-\gamma & 0\\ 0 & \gamma & 1 \end{array}\right]$$ (2)

The model developed in this paper incorporates asymptomatic infection (IA), quarantine (Q vs NQ), mortality (D), and differentiates symptomatic people by disease severity (ISM for mild vs ISS for severe). It can be described by Eqs (3)–(5).

$${state}_{t+1}^{i}=P_{t}^{i}\times {state}_{t}^{i}$$ (3)

where

$${state}_{t}^{i}={\left[{SQ}_{t}\;{SNQ}_{t}\;{IAQ}_{t}\;{IANQ}_{t}\;{ISM}_{t}\;{ISS}_{t}\;R_{t}\;D_{t}\right]}^{T}$$ (4)

$$P_{t}^{i}=\left[\begin{array}{cccccccc} 1-\sigma & w_{t}^{i} & 0 & 0 & 0 & 0 & 0 & 0\\ \sigma & 1-\beta g_{t}^{i}I_{t}^{NQ}-w_{t}^{i} & 0 & 0 & 0 & 0 & 0 & 0\\ 0 & 0 & 1-\mu -\gamma_{1} & h_{t}^{i} & 0 & 0 & 0 & 0\\ 0 & \beta g_{t}^{i}I_{t}^{NQ} & 0 & 1-\mu -h_{t}^{i}-\gamma_{1} & 0 & 0 & \rho g_{t}^{i}I_{t}^{NQ} & 0\\ 0 & 0 & \mu & \mu & 1-\gamma_{2}-\tau & \gamma_{3} & 0 & 0\\ 0 & 0 & 0 & 0 & \tau & 1-\gamma_{3}-\delta & 0 & 0\\ 0 & 0 & \gamma_{1} & \gamma_{1} & \gamma_{2} & 0 & 1-\rho g_{t}^{i}I_{t}^{NQ} & 0\\ 0 & 0 & 0 & 0 & 0 & \delta & 0 & 1 \end{array}\right]$$ (5)

in which $I_{t}^{NQ}=\frac{IANQ_{t}}{SNQ_{t}+IANQ_{t}+R_{t}}$ is a shorthand for convenience. Equivalently, the above expansion can be considered as starting with the standard SEIR model, and replacing the exposed state by the IA states. Both approaches have been considered by existing literature and result in similar model frameworks that best describe COVID-19 [21, 22, 24]. Detailed explanation for each term in this transition matrix is provided in the S1 Appendix section A1.1.

The transition matrix embeds two key internal policy parameters. The social distancing parameter, $g_{t}^{i}$, is a factor between 0 and 1. When $g_{t}^{i}=0$, The transition from SNQ state to IANQ state is shut down, meaning that everyone is in self-quarantine, and no new infection could happen. When $g_{t}^{i}=1$, the transition rate, $\beta g_{t}^{i}I_{t}^{NQ}$, is very similar to the transition rate in the standard SIR model, i.e. $\frac{\beta I_{t}}{N}$, since $I_{t}^{NQ}$ is the share of infected people in the local community conditioning on the subpopulation not in quarantine or hospitalized. A smaller value of $g_{t}^{i}$ corresponds to a constrictive social distancing policy measure, and hence a smaller transition probability from SNQ state to IANQ state. The contact tracing parameter, $c_{t}^{i}$, is also a factor between 0 and 1. The transition rate from IANQ to IAQ, $h_{t}^{i}$, is an increasing function of $c_{t}^{i}$ with no intercept, i.e. $\partial h_{t}^{i}/\delta c_{t}^{i}$>0 and $h_{t}^{i}=0$ when $c_{t}^{i}=0$. The other two internal policy parameters related to self-quarantine for susceptible people are less essential to answering the research question, and thus we discuss them in the extension section.

We next model travelers across the areas. We allow the epidemiological patterns of the travelers to differ from that of the overall population at their departure area. This means that the distribution of ${travel}_{t}^{ji}$ could be different from ${state}_{t}^{j}$. For example, in S1 Appendix section A2.2, we show empirical evidence for the possibility that the fraction of IANQ people among the travelers could be amplified when some of them self-select to travel to a low risk area. We also expose the travelers to a higher infection rate due to the lack of social distancing on their way to the destination. The transition matrix for travelers on the road in period *t* is $P_{t}^{ji}$. The government at the destination area has three policy measures for inbound travelers. It may impose traveling bans by adjusting the number of inbound travelers $N_{t}^{ij}$. The government may also choose to quarantine the inbound travelers, with the daily transition matrix in quarantine period being $\tilde{P}$. The government may test them, with the testing transition matrix being ${\hat{P}}_{t}^{i}$. The population in an area equals to local population, minus outbound travelers, and plus all inbound travelers coming out of quarantine. We convert Eq (3) to the following equation:

$${state}_{t+1}^{i}=P_{t}^{i}\left({state}_{t}^{i}-\sum_{j\neq i}{travel}_{t}^{ij}\right)+\sum_{j\neq i}\sum_{x\;s.t.q_{t-x}^{i}=x\&x\geq 0}{{\left({\hat{P}}_{t-x}^{i}\right)}^{r_{t-x}^{i}}(\tilde{P})}^{x}P_{t-x}^{ij}{travel}_{t-x}^{ji}$$ (6)

More detailed explanations for the model specification are provided in the S1 Appendix section A1.

**Fig 1:**
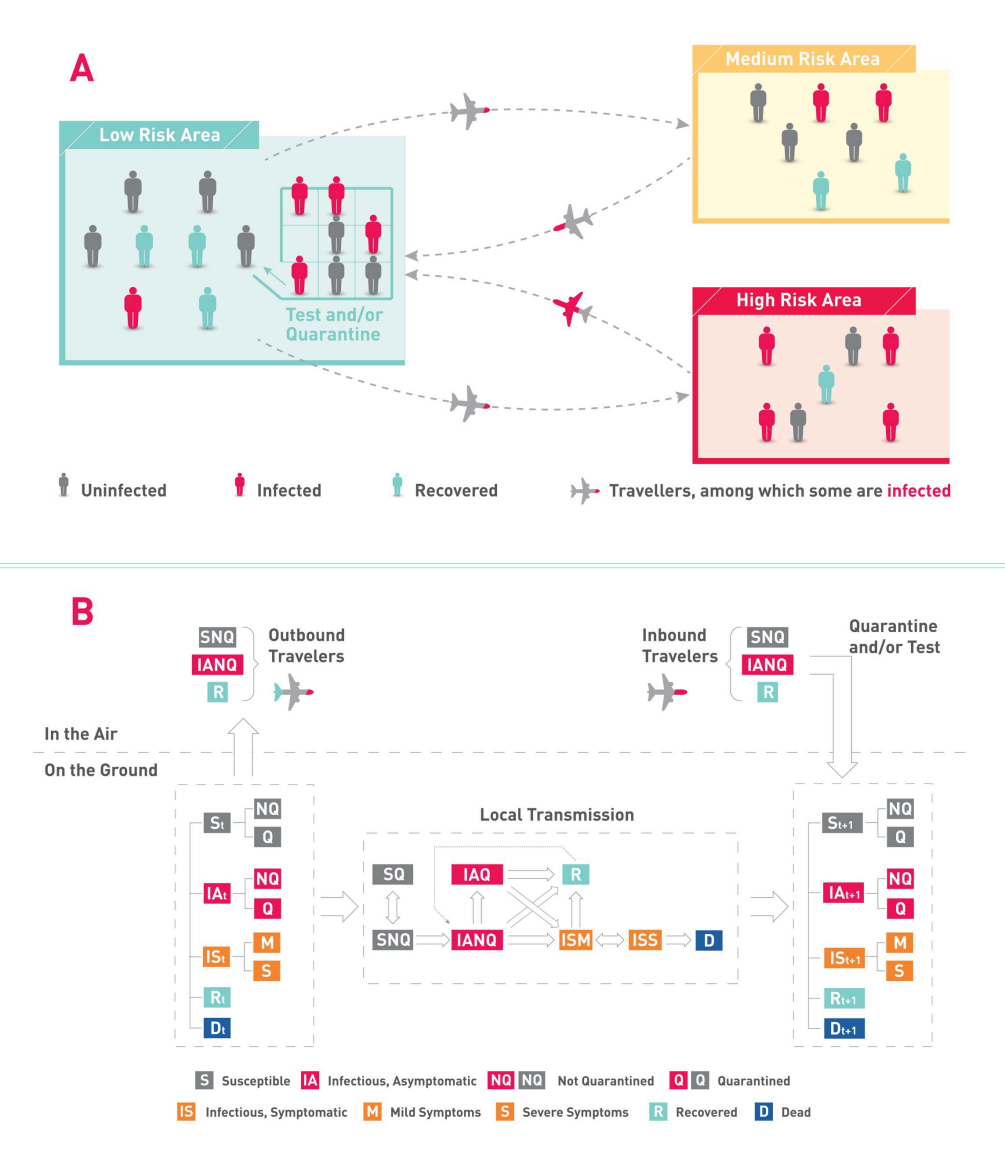
Schematic illustration of the model framework. (A) Pandemic outbreaks affecting three areas differently, with travelers between a low risk area and medium/high risk areas. The low risk area determines external policy including tests and/or quarantine to contain the virus outbreak while reopening the area. (B) One area in a pandemic, facing heterogeneous inbound and outbound travelers, local transmission with quarantine options.

Fig 1 shows the model framework, in which Fig 1A presents the interaction across areas, and Fig 1B presents the disease spreading from one area’s perspective with in-and outbound travelers. The model intends to support policymakers by providing quantitative information on the magnitude of the effects of each potential single or combined policy intervention. For example, later in the paper, we show that dynamically adjusting the numbers of travelers from areas with medium and high epidemic risk to keep the daily new imported cases as a constant can effectively reduce the public health effect of inbound travelers on local people.

The model framework should be interpreted as a general model with multiple epicenters of a pandemic and travelers across the areas. The generality of the mathematical model is mostly reflected in the setup of the transition matrix. The transition matrix for each area can characterize diseases that follow several typical epidemic models in addition to SIR. For example, when the reinfection rate *ρ* is positive, the model embeds an SIS model. When the reinfection rate is the same as the transmission rate, i.e. *ρ* = *β*, the model corresponds to the SI model. When we allow the infection probability to differ by the asymptomatic patient’s stage in the incubation period, as stated in a remark in the S1 Appendix section A1.1, the special case in which the early stage of incubation is noninfectious corresponds to an expanded SEIR model. The SQ state is similar to the maternally-derived immunity state in compartmental epidemic models, with a rate of becoming susceptible (*σ*). The deceased state (D) allows mortality to be included or excluded by specifying the mortality probability (*δ*). The flexible transition matrix allows our model to be applied to other recent epidemics that follow compartmental epidemic models such as H1N1 (the SIR model [26, 27]), the Ebola virus disease (the SEIR model [28]), and the seasonal flu in the U.S. (the SEIRD or SIRS model [29, 30]). On the other hand, note that the model focuses on government policy evaluation with the assumption of a homogeneous population. This assumption can be relaxed to capture the heterogeneous epidemiological characteristics of people with distinct demographic features. The extended model will be able to predict the policy effects on specific subpopulations. For example, one may combine the model presented in this paper with the single-region multiple-age group model discussed in [31] to study the effect of policies on each cohort.

As of August 2^nd^, 2020, at least 177 countries have issued international travel bans to restrict virus spread across the borders [24]. Meanwhile, 164 countries implemented internal policies such as closing schools and public transportation, prohibiting social gatherings to reduce the chance of infection [32]. South Korea, the U.S., and China introduced contact tracing through mobile phones to track down people who may have been infected by the newly confirmed patients [33, 34]. We translate these policies into modeling features as follows. An area with low epidemic risk (denoted by *L* in the superscript) faces travelers from a medium risk area (denoted by *M*) and a high risk area (denoted by *H*). We allow the low risk area to choose separately for each foreign area the inbound travel capacity quota and the ratio between traveler capacities of inbound and outbound traffics. The low risk area may quarantine the inbound travelers for $q_{t}^{L}$ days and test them for $r_{t}^{L}$ times when they are quarantined. The type II error (probability of a false negative outcome) of one test is *θ*. We assume quarantined people cannot infect others or be infected. We also allow the low risk area to choose its social distancing policy parameter *g*, which is positively correlated with the local infection rate, and its contact tracing policy parameter *c*, which determines the probability that an infected asymptomatic person will be quarantined when the person who infected him becomes a confirmed patient. All of the above policy parameters can change over time. Given this general model framework, many other factors are flexible and are at the discretion of the specific scenario to which the model is applied.

We calibrate the model using approximated population distribution from three areas featuring each type [35–37]. In the model calibration, we assigned realistic values to the parameters and probability distributions based on public information to the best of our knowledge and general intuition to the best of our ability. We only state their values in the text, leaving the mathematical calculation and reasoning behind these assignments in the S1 Appendix sections A2.4–2.5. We assume the populations of the three areas are the same. To illustrate the model implication on COVID-19, we assume the average incubation period is 5.2 days [38], the average duration of illness is 14 days [39], and the infection fatality rate is 0.6% [40]. In the baseline model, the test accuracy is 80% [41], all travelers are quarantined for 7 days and are tested twice. The number of travelers on each route between areas is set to be 0.005% of the local population in the initial state of the model, which is close to the March 2020 scenario in the U.S [42]. For internal policies, the baseline model assumes heterogeneous internal policy factors. It assigns the low risk area with a perfect contact tracing system, while the contact tracing mechanism is only 50% effective in the medium and high risk areas. It allows the social distancing parameter to be larger for higher-risk areas. For travelers, the model assumes that only asymptomatic people who are not quarantined can exit any area. We apply an amplification function to characterize the fact that the fraction of infected people is much higher among the travelers than in the population that the travelers’ sample is drawn from. An example of the amplification function is introduced in S1 Appendix section A2.2. We also apply the feature that susceptible travelers suffer from a higher infection rate from exiting the departure area to entering the destination area due to the lack of social distancing. The daily flows of inbound and outbound travelers do not have to be the same, but in order to make clear inferences from the model calibration and its simulation results, we assume the daily flow of travelers between two areas is the same across directions for the symmetry of inter-area policies. Interested readers can relax this assumption by specifying the traveler ratio $N_{t}^{ij}/N_{t}^{ji}$ in the computer program to have $N_{t}^{ij}\neq N_{t}^{ji}$.

In the baseline model specification of the low risk area, we set the contact tracing probability to 1 and the social distancing parameter to 0.25. We set the daily flow of travelers from every other area to be 0.005% of the low risk area’s local population, which is close to the March 2020 scenario in the U.S. [42]. We assume the medium- and high-risk areas do not quarantine or test travelers coming from the low risk area. We set the social distancing parameter to 0.7 for the medium risk area, and 1 for the high-risk area. In the illustration, we define daily new cases as the number of all newly infected local people plus the number of infected people from travelers who come out of quarantine on that day. In the extension section, we discuss alternative definitions of new cases.

**Fig 2:**
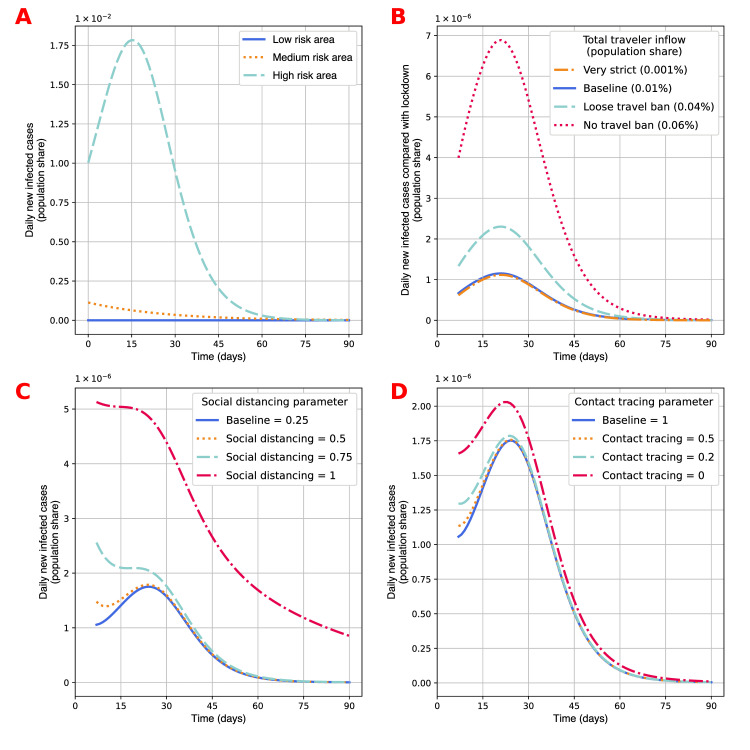
Effect of the internal and external policies on the virus outbreak. The low risk area in the baseline model setting: social distancing parameter *g*^*L*^ = 0.25; contact tracing probability is *c*^*L*^ = 1; travel capacity quota, characterized by the ratio between the number of inbound travelers from area *i* to the low risk area and the local population in *L*, is *N*^*iL*^ = 0.005% for *i* = *M*, *H*; the default duration of quarantine is *q*^*L*^ = 7; the number of tests taken by the travelers and new to-be-confirmed cases is *r*^*L*^ = 2; the accuracy of the test is 1−*θ*^*L*^ = 0.8. For more detailed information on the notation and parameter values, please see Table 1 and S1 Appendix sections A2.4–2.5. (A) Daily new infected cases in the three areas when all areas implement lockdown, i.e. we let *N*^*ij*^ = 0 for all *i*,*j*∈{*L*, *M*, *H*}, showing the heterogeneous phases of the COVID-19 pandemic in different areas. The internal policies in the medium and high risk areas are *g*^*M*^ = 0.7, *g*^*L*^ = 1, and *c*^*M*^ = *c*^*L*^ = 0.5. The numbers of newly infected cases differ significantly in magnitude, so the number of new cases in the low risk area seems very small. It is 1.2×10^−6^ on day 1, and decreases monotonically from then on. (B) Effect of relaxing the travel capacity quota from a very strict travel ban (i.e. each *N*^*iL*^ = 0.0005%) to completely free entry (*N*^*iL*^ = 0.03%). Displayed statistics are the population share of daily new infected cases in each scenario minus the population share of daily new infected cases in the lockdown scenario (*N*^*iL*^ = 0). To visualize the effects of travel bans without being covered up by other external policies, the scenarios in this subfigure do not screen the inbound travelers, i.e. no tests (*r*^*L*^ = 0) and no quarantine (*q*^*L*^ = 0). (C) Effect of relaxing social distancing from baseline (*g*^*L*^ = 0.25) to the scenario without social distancing (*g*^*L*^ = 1). (D) Effect of relaxing contact tracing from baseline (*c*^*L*^ = 1) to the scenario without contact tracing (*c*^*L*^ = 0).

We first visualize in Fig 2A the time trend of daily new cases in those areas if they all resort to lockdown by blocking cross-area traffics. The lockdown policy deactivates all external sources of new cases and external policies, but does not affect any internal policy settings specified in the notes of Fig 2. It shows the distinction among the three areas in terms of initial population status and the speed of virus transmission within the area. In this circumstance, the number of daily new infected cases in the low risk area drops to below 1×10^−8^ of the population by day 19 in the model simulation. The medium risk and high risk areas have more new infections in the initial states of the model simulation, and relaxed internal policies create more infection.

## Results and discussion

### Roles played by the internal and external policy interventions

In Fig 2B, we present the additional daily new local cases in the low risk area as we gradually reopen the area by increasing its traveling capacity. To separate the effect of travel capacity from the effects of tests and quarantine for inbound travelers, we set the numbers of tests and quarantine days for travelers upon arrival to zero. The statistics we present are the number of new infections in the local population in each scenario minus the new infection in the lockdown case shown in Fig 2A. It shows that increasing the total number of inbound travelers causes a minor outbreak in the local area. When the traveler inflow is 0.06%, the scenario is comparable with the pre-pandemic scenario in many countries [43]. This shows that the absence of travel bans leads to a new round of epidemic outbreaks with 0.0007% of the local population being newly infected cases in one day at peak. This figure provides quantitative evidence for the belief that, keeping testing and quarantine policies towards inbound travelers unchanged, opening the border to areas with higher epidemic risk would result in more infection in the domestic area. In the next section, we will show the percentage contribution of imported cases in different policy environments. Then we test internal reopening strategies on the baseline model, by reducing the social distancing parameter among local people in this area with very few local cases (Fig 2C), and loosening the contact tracing strength to allow more infected people in the local area to stay out of quarantine (Fig 2D). Fig 2C shows that relaxing social distancing policy causes more infection. Lifting the social distancing policy measure completely boosts the new infected cases by folds at the peak, and leads the epicenter to unrest after day 75, unlike other scenarios would. Fig 2D shows that relaxed contact tracing policy amplifies the small outbreak that travelers would cause. Without the contact tracing policy, the low risk area also generates another minor outbreak with 2.05×10^−6^ new infection on the worst day. Cumulatively, by day 60 in the model simulation, the absence of the social distancing policy and contact tracing policy could cause 1.8×10^−4^ and 1×10^−4^ additional infected cases, respectively. We find that both internal and external policy interventions affect virus spreading in the low risk area, and the magnitudes of their effects are dramatically different. The quantitative effects of the social distancing policy and travel bans are comparable, and they are about 10 times the effect of contact tracing policy. The model implies that relaxation in the external policy towards reopening must be complemented by a constrictive internal policy, and vice versa. Though both policy directions are important in containing the epidemic, social distancing policies and some scale of travel restrictions should have higher priority.

### Flexible and composite policies towards inbound travelers

**Fig 3:**
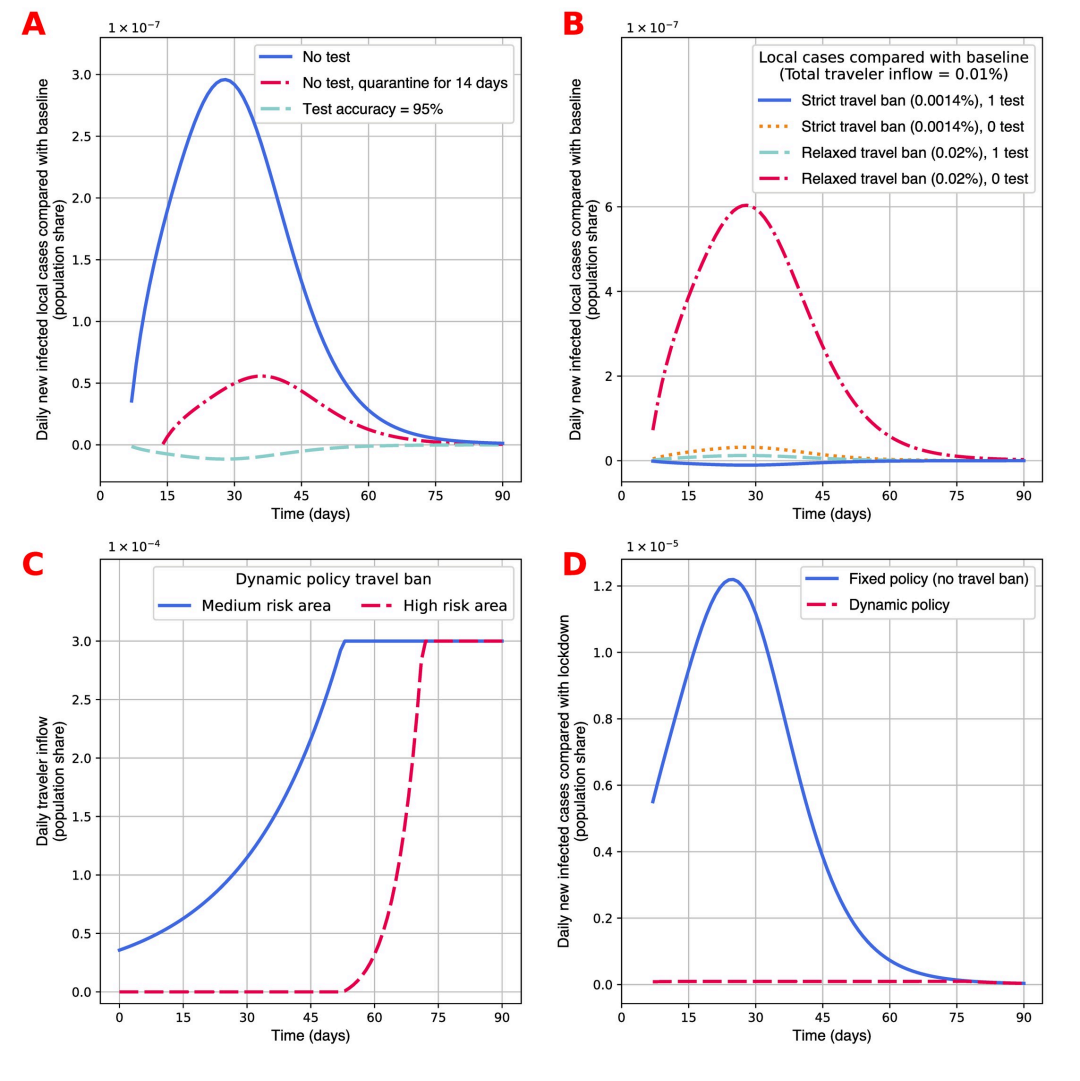
Effect of specific external policies. (A) Effects of three external policy schemes are presented. The effects are relative to the baseline model described in Fig 2. The three policy schemes are no test (*r*^*L*^ = 0 and *q*^*L*^ = 7), no test accompanied by prolonged quarantine period (*r*^*L*^ = 0 and *q*^*L*^ = 14), and test accuracy improves to 95% (*θ*^*L*^ = 0.05). (B) Effects of four external policy schemes involving travel bans and testing are presented. The effects are relative to the baseline model described in Fig 2. The strict travel ban is a 86% reduction in travel capacity quota (*N*^*iL*^ = 0.0007%), which is equivalent to the policy in China from March 29, 2020, onward [44]. The relaxed travel ban simply doubles the travel capacity quota in the baseline, i.e. *N*^*iL*^ = 0.01% for each *i*. (C) Implementation of a dynamic policy that sets a quota on the traveler capacity of *N*^*iL*^ = 0.03% for each *i*, and another quota that the population share of infectious inbound travelers cannot exceed 0.00001%, i.e. the IANQ element of $P_{t}^{ji}\times {travel}_{t}^{ji}<1\times 10^{-7}$. This is equivalent to 140 new imported cases each day for the population of mainland China. The statistics shown are the daily traveler inflow by area type determined by the dynamic policy. (D) The performances of the dynamic policy and the fixed policy are presented. The statistics shown are daily new infected cases of each policy minus the number of cases of a lockdown policy with *N*^*iL*^ = 0, and a fixed policy with *N*^*iL*^ = 0.03% for each *i*, which is equivalent to the scenario without a travel ban. For all scenarios, the number of tests is 0 (*r*^*L*^ = 0) and the duration of quarantine for inbound travelers is 7 days (*q*^*L*^ = 7).

The model enables the estimation of the marginal effects of each component of the external policy interventions. In Fig 3A we present the effects of adjusting the traveler’s duration of quarantine, the number of tests, and the accuracy of the tests based on the baseline model specification. We want to focus on the externality of external policies on internal epidemic conditions, so the statistics shown in the figure are the number of new infected cases among the local population in each scenario minus the number from the baseline scenario. Test accuracy only affects the virus containment in two ways. One is to identify IANQ people who were traced to have close interaction with confirmed cases, and the other is to accurately identify infectious inbound travelers who have not developed symptoms when they come out of quarantine. Both channels are fairly minor, so the overall effect of increased test accuracy is much smaller than the screening measures on the travelers. The contribution of testing is very substantial. If the low risk area does not test the travelers, the number of cases would surge by about 3×10^−7^ at peak. Extending the quarantine period can compensate for the lack of testing. The number of local cases drops by about 80%, as most infected people would have developed symptoms within 14 days, resulting in infected travelers being hospitalized before they are released from the quarantine. This comparison leads us to believe that testing and quarantine can help with reducing the number of cases, especially when the epidemic condition of the travelers’ departure areas is significantly worse than the local condition. Considering the complementarity between quarantine and tests, the negative impact of less testing can be offset by extending quarantine, and the effort to improve the test quality may not pay off as well as other behavioral policies.

In Fig 3B we present the interplay between travel bans and the screening on inbound travelers. When the screening involves testing and quarantine, the low risk area can have fewer local cases as it reopens the border. This is because the outbound travelers may have some IANQ people, while the IANQ people among the inbound travelers would have been identified by the quarantine and testing, and thus would not be blended into the local population. However, holding travel ban setting constant, the lack of testing (or quarantine) would always result in more local cases. As Fig 3B shows, if the low risk area admits more travelers, it would pay a higher price in terms of additional cases if its government did not pair a relaxed travel ban with a constrictive testing and quarantine mechanism.

We illustrate a dynamic policy in Fig 3C and its performance in Fig 3D. In the dynamic policy, the reopening strategy taken by the low risk area is to set a quota on the number of infected asymptomatic inbound travelers. In this strategy the low risk area would estimate the fraction of infected travelers from each of the other two areas, and choose the traveling capacity with them accordingly, prioritizing traffic from areas with relatively low risks. The upper bound of traveler inflow is 0.003% for each area, equivalent to the scenario without a travel ban or the pandemic. This means the fixed policy is the same as the most relaxed scenario shown in Fig 2B, while the dynamic policy can choose any specific quota up to the same level as the fixed policy. The testing and quarantine policies are the same for the two policy schemes. Fig 3C shows the specific implementation of the policy. The low risk area first bans travel from the high risk area completely, and allows very few travelers from the medium risk area to enter the border. As the medium risk area gradually contains the virus outbreak, the low risk area expands the traveling quota accordingly. By day 50, travelers from the medium risk area do not face any quota, while no traveler from the high risk area is allowed to enter. By that time, the epidemic risk in the high risk area is much lower than its most devastating period, so the low risk area then reopens its border slowly, and fully reopens by day 70. In Fig 3D, the gap between the time trends in daily new infection in the dynamic versus the fixed policy, relative to the lockdown scenario, shows its role in protecting the local people in the low risk area. This dynamic reopening policy performs almost as well as the lockdown policy while allowing some travelers to come in. This policy scheme effectively disentangles the correlation between the epidemic condition in the low risk area and the conditions in the departure areas of its inbound travelers.

We find that the effects of specific components of external policies in the reopening strategy vary in magnitudes. Flexible combinations of these potential policies are key to areas highly constrained in testing ability, medical capacity, or government budget in public health. When the composition of policies is well designed, reopening can cause much fewer local infections.

## Extensions

We discuss five extensions of the model to enhance interpretation of the model framework, and shed light on how to use the model. The first two extensions explore alternative approaches to consider the number of new cases and their inferences. The third extension concerns the model’s potential in incorporating reinfection. The fourth extension uses the model framework to evaluate self-quarantine policies. And the last subsection illustrates the model usage in calculating the government spending on travel-related policy implementation and infection cases.

### Specific definitions of new cases

**Fig 4:**
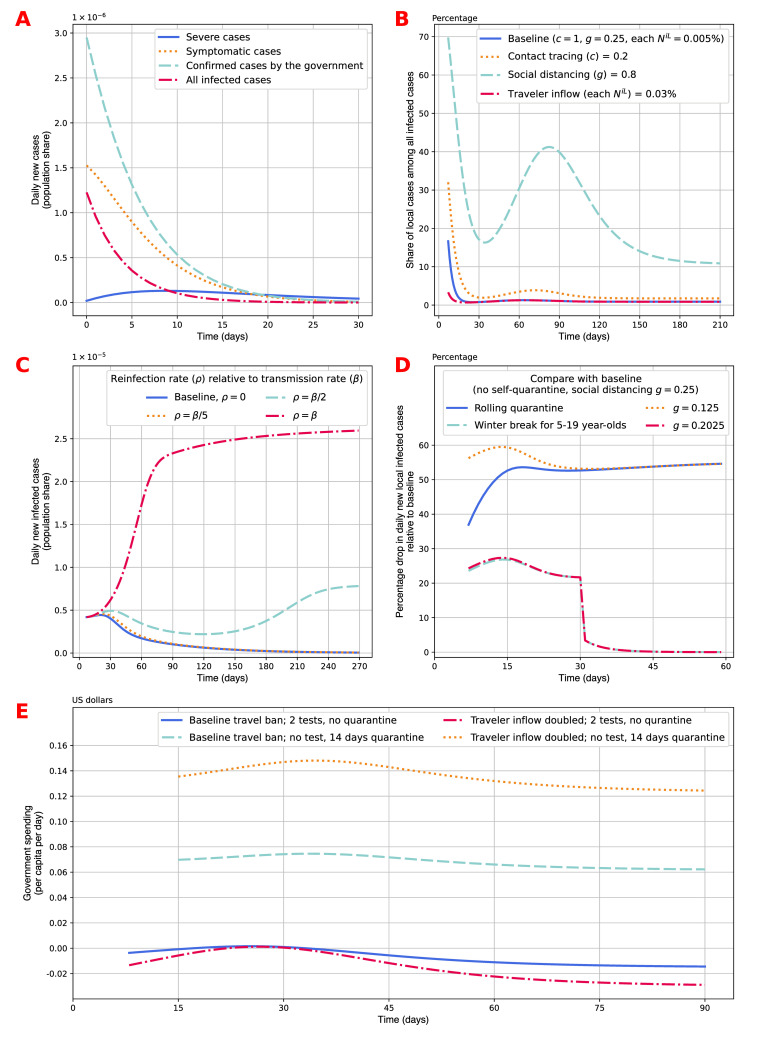
Extended interpretations of the model. (A) Numbers of new cases with four different definitions are presented. Severe cases count new entry into the severe case (ISS) state. Symptomatic cases count new entry into the mild symptom (ISM) state. Confirmed cases by the government count new asymptomatic entry into quarantine (IAQ) and new symptomatic cases (ISM). All infected cases count all new entries into the asymptomatic state (IANQ). (B) Effects of internal and external policies on the share of local cases among all new infections are presented. The contact tracing, social distancing, travel policies change one of the policy parameter values as labeled in the legend and keeps all other values consistent with the baseline model. (C) Effects of reinfection rate values are presented. The value of the reinfection rate for recovered people is relative to the value of the transmission rate of susceptible people. The baseline model assumes recovery confers long-term immunity, i.e. *ρ* = 0. When 0<*ρ*<*β*, the model embeds an SIS model. When *ρ* = *β*, the model embeds an SI model. (D) Effects of self-quarantine policies are presented. The effects presented are the percentage drop in daily new local infected cases relative to the baseline specification described in Fig 2B. The rolling quarantine policy randomly selects 1/14 of the population and asks them to self-quarantine for 14 days. In this policy scheme, about 50% of all susceptible people are in self-quarantine. The policy labeled as “winter break for 5–19 year-olds” asks all school-aged children to take a 30 days winter break and self-quarantine during this period. This policy scheme quarantines 19% of the susceptible population in the first 30 days of the model simulation. The two social distancing policies are used to compare the effects of different internal policies. (E) Government spending related to travelers is defined as the total of quarantine, testing, and treatment costs, minus the economic gain from travelers who are released from quarantine. Daily costs per capita over time are presented for four scenarios: baseline travel ban (each *N*^*iL*^ = 0.005%) with testing requirement (*r*^*L*^ = 2, *q*^*L*^ = 0), baseline travel ban with quarantine requirement (*r*^*L*^ = 0, *q*^*L*^ = 14), relaxed travel ban (each *N*^*iL*^ = 0.01%) with testing requirements, relaxed travel ban with quarantine requirement.

In all previous sections of this paper, we have used the term “all infected cases”. It means that we count everyone who becomes infected in a given day as a new case. People have to become infected before they were identified as infectious people and were quarantined, so the number of new infections is the number of people who become IANQ on that day. There can be other definitions of new cases. The usual term used by the government is “confirmed cases”, which corresponds to everyone who either becomes symptomatic on that day, or was tested positive by the government, and hence was quarantined, even though he or she did not show any symptom. So confirmed cases count people who become IAQ or ISM on that day. In addition, we can also count new cases from the perspective of the demand for healthcare. The number of symptomatic cases corresponds to the demand for hospital beds or nurses. And the number of severe cases is in proportion to the demand for doctors or ICU beds. Fig 4A shows the relative sizes of the four ways to count new cases. At the beginning of the virus containment period, the numbers of new infections, new confirmed cases, and new symptomatic cases all exceed the number of severe cases. When we see more new severe cases than new infections, it implies that the containment policies start to take effect. Then when we fewer new symptomatic cases than new severe cases, it means we are in the last phase of the outbreak and the virus is almost fully contained.

### Local and imported cases

As we presented in Eq (6), the number of new cases in an area with travelers on a given day is the sum of two terms: imported cases, which is the sum of all infected people among the inbound travelers who come out of quarantine on that day; and local cases, which is the number of susceptible (or recovered, if reinfection is possible) people who become infected on that day. Counting the new infected cases from these two sources separately can help us understand the relative effect of internal and external policies, as well as the boundary that these two types of policies may take effect. Fig 4B presents the share of local cases among all infected cases in the baseline specification. It also presents the same statistic for the scenarios with a more relaxed contact tracing policy, social distancing policy, and traveling control. When the internal policy is more relaxed, local cases contribute a larger fraction to the overall new infected cases in the area. Relaxing external policies, on the other hand, expands the share of imported cases among all new infected cases. The curves for the baseline scenario and the relaxed external policy scenario converge after day 24 and stabilize at 0.88% after day 120, meaning that the fraction of imported cases does not depend on the travel ban after the internal virus outbreak is well-contained. In the scenarios with relaxed contact tracing and social distancing policies, the shares of local cases experience a small outbreak between day 60 and day 120, then stabilize at 1.7% and 10.9%, respectively. The two scenarios with relaxed internal policies never converge with the baseline scenario. This observation tells us that internal policy measure decides the fraction of local cases among all new infections in the steady state.

### Reinfection after recovery

Some reinfection cases have been reported by the CDC of the U.S., while the cases remain rare [45]. In Fig 4C, we present the population share of daily new infected cases for the scenarios from complete immunity (i.e. Baseline model, in which *ρ* = 0) to no immunity (*ρ* = *β*). The disease would cause more new cases if the reinfection is high. If recovery does not confer immunity at all, i.e. *ρ* = *β*, the daily count of new infected cases increases rapidly in the first 3 months, and gradually stabilizes at 0.0025% in the long term. When *ρ* = *β*/2, the daily new infected cases in the steady state is 0.0008%. On the other hand, the scenario with *ρ* = *β*/5 is very similar to the baseline scenario with *ρ* = 0, as both specifications result in new cases drop down to below 1×10^−7^ around day 230. This figure shows that the speed in which the virus outbreak cools down depends on the ratio between the rates at which susceptible and recovered people become infected. It would be an interesting future research topic to find the formula for the threshold of *ρ* below which the daily new infected cases would converge to zero.

### Self-quarantine policy for susceptible

Self-quarantine or isolation is often mentioned in the media yet lacking quantitative measures for its effect [46]. The transition matrix allows for modeling self-quarantine by including an SQ (susceptible-quarantine) state. To illustrate the use of this state, we simulate two types of self-isolation policies. The first policy is a rolling quarantine scheme, in which the government asks 1/14 of the susceptible population to self-quarantine for 14 days. On average, this is equivalent to asking half of the population to self-isolation each day from day 14 onward. The second is a policy that asks all school-aged children (5 to 19 years old) to stay at home during the 30-day winter break from day 0 to day 29, then nobody is in self-quarantine from then on. This is equivalent to quarantining 19% of the population [47]. Fig 4D presents the effects of the four different policy changes by showing the percentage drop in daily new local infected cases compared with the baseline model with social distancing parameter *g* = 0.25 and no self-quarantine. Positive percentage drops in cases show that both self-quarantine policies help contain the virus spread. The rolling quarantine policy and the school-aged children quarantine policy reduce the number of cases by 55% and 28% at peak, respectively. In Fig 4D, we also compare the effect of the schemes with social distancing policy with the same percentage drop in social interaction. This corresponds to the social distancing parameter reducing to *g* = (1−50%)×0.25 = 0.125 and *g* = (1−19%)×0.25 = 0.2025, respectively. The performance of the social distancing policies is significantly better than the rolling quarantine policy in the first month, and the effects converge afterward. The performances of the school-aged children quarantine policy and the comparable social distancing policy are similar. This comparison shows the importance and effectiveness of social distancing relative to other virus containment policy measures.

### Government spending analysis of external policies

The model also allows us to calculate government spending associated with travelers, which are particularly important for countries in which the tourist industry makes a crucial contribution to their economy and tax revenue. This analysis at least includes the costs of testing kits, medical treatment, quarantine, and all the direct and indirect consumption and trades introduced by the travelers. In the calibration, we assume the test kits ($51) [48], medical treatment for patients with severe symptoms ($34,223) [49], medical cost for patients with mild symptoms ($3,045) [50], and quarantine ($62) are all covered by the government [51], and the average tax revenue generated from one international traveler is around $314 [42]. The model simulation presented in Fig 4E shows that for each fixed travel ban specification, replacing 2 tests by a 14-day quarantine requirement increases government spending. A relaxed travel ban would increase the number of local new cases induced by infected travelers who did not show symptoms during the quarantine and were not detected by the imperfect testing. Interestingly, when the screening on inbound travelers is strict, having two tests, allowing more travelers to come in generates a small economic gain. But when the screening is loose, the economic consequence of relaxing travel restrictions is large. Moreover, the additional cost caused by the lack of testing increases proportionally with the number of inbound travelers. We conclude that internal and external policies interplay in determining the government spending in the reopening process, and that a simultaneous relaxation of these interventions causes a sizable shock to the government. Note that this calculation needs to incorporate the effects of social distancing and travel bans on GDP if interested readers want to conduct a comprehensive economic analysis.

## Discussion

Studying the effect of travelers on local COVID-19 outbreaks is much needed to design an area’s reopening strategy. Addressing inbound travelers and the epidemic externality associated with them are a combination of public health, economics, and political research questions. In this paper, we studied the epidemiologic and economic perspectives of policies on travelers and local people to provide quantitative evidence for policy discussion. We presented a dynamic model of three areas in heterogeneous phases of the COVID-19 outbreak with travelers across the areas. The model features social distancing, contact tracing, and self-quarantine as internal policies, and travel restriction, quarantine, and testing as external policies. Calibration using estimated parameters from the COVID-19 data shows simulated outcomes of policy changes on local epidemic conditions.

We found that internal and external policies toward reopening complement each other, while simultaneous relaxation of both policies would result in considerable health risk to the local population. Specific external policies partially substitute each other, and a dynamic combination of them can generate effects as good as extreme choices such as a lockdown. Reopening strategies also create diverse impacts on the economy. When we solely consider the impact of travelers on the economy, easing policies towards travelers and local people at the same time would lead to huge public health costs exceeding economic gains.

The model illustration has some limitations that may be expanded by future research. For example, the calibration assumed the three areas to have the same population size for simplicity and convenience in the presentation of the results. We give instructions for implementing heterogeneous population sizes across areas in the computer program. In such situations, the users of the model should focus on modeling the ratio between the numbers of inbound and outbound travelers, instead of the population shares of them.

Much further research and policy questions may be answered by extending the model we developed. One might solve for the optimal reopening strategy from the public health or economic perspective while keeping the costs in the other aspect constant. One might easily build a testing capacity constraint on the model to study the optimal allocation of test kits among travelers and domestic asymptomatic cases. One might also attach macroeconomic and international trade components to the model to better characterize and include the internal GDP reduction caused by social distancing in the overall cost-benefit analysis. We strongly encourage policymakers and the general public to make use of the computer program that simulates our model and configure the model setting to their own context to enhance understanding of the current and upcoming scenario.

## Supporting information

S1 Appendix (DOCX)
