# Supplementary material for: A dynamic pandemic model evaluating reopening strategies amid COVID-19
Source: PLoS One. 2021 Mar 26;16(3):e0248302. doi: 10.1371/journal.pone.0248302 (PMC7996987; doi:10.1371/journal.pone.0248302)
Supplement: S1 Appendix — (DOCX) [file pone.0248302.s001.docx]

**S1 Appendix**

**A1 An epidemic model with multiple areas, inter-area traffic, and policy interventions**

We develop the model to formalize the mechanism of a global pandemic in which different areas are in heterogeneous stages of the epidemic outbreak, accounting for the flow of traffic across areas and the potential public health and foreign policies that local and national government can implement in response to constantly changing domestic and international scenarios. We propose a general model framework in this section, then calibrate the model by specifying parameters values and functional forms in the next section.

In this section, we first describe the domestic virus spreading and the government’s internal policy for the local community. Next, we describe the traveler’s epidemiological characteristics and virus spread among them. Then we describe the government’s external policy towards travelers. Lastly, we summarize the model framework and highlight the innovative components of the model.

**A1.1 Domestic virus spreading and policies**

The model includes multiple areas. Time is discrete. The population of area $i$ in the initial state is $N^{i}$. The domestic scenario, or state, of area $i$ is summarized by

${state}_{t}^{i}=N^{i}\times\left[ pr\left( \mathrm{SQ} \right),pr\left( \mathrm{SNQ} \right), pr\left( \mathrm{IAQ} \right), pr\left( \mathrm{IANQ} \right), pr\left( \mathrm{ISM} \right), pr\left( \mathrm{ISS} \right), pr\left( R \right),pr(D) \right]^{T}$ (7)

which is the population distribution of its people among the 8 compartments summarized in Table A1.

**Table A1. Compartments of the model**

| **Compartment** | | **Abbreviation** |
| --- | --- | --- |
| **Health status** | **Social status** |  |
| Susceptible | Quarantined | SQ |
| Susceptible | Not quarantined | SNQ |
| Infectious, Asymptomatic | Quarantined | IAQ |
| Infectious, Asymptomatic | Not quarantined | IANQ |
| Infectious, Mildly Symptomatic | Assumed to be isolated | ISM |
| Infectious, Severely Symptomatic | Assumed to be isolated | ISS |
| Recovered | Assumed to be not quarantined | R |
| Dead | - | D |

We denote the $k$-th element of ${state}_{t}^{i}$, i.e. $pr\left( \mathrm{compartment} k \right)$ at time $t$, by ${state}_{t}^{i,k}$.

The pattern of the virus spread among local people is characterized by an area-, time-, and state-specific transition matrix $P_{t}^{i}$ presented in equation (5). We denote the entry in $P_{t}^{i}$ that corresponds to $pr\left( \left. j_{t+1} \right|i_{t} \right)$ by $P_{t}^{i} \left( j_{t+1},i_{t} \right)$. We explain all the non-zero terms in the transition matrix as follows. Please note that for terms that are defined as functions of other parameters, we list the conditions that those functions should satisfy in this section, and specify functional forms in the Calibration section. We also assign specific values to the parameters in the Calibration section.

- People who are in state SQ in period $t$ are people who stayed in self-quarantine.
  - They may come out of self-quarantine at a rate of $P_{t}^{i} \left( \mathrm{SNQ}_{t+1},\mathrm{SQ}_{t} \right)=\sigma$,
  - Or stay in self-quarantine, i.e. $P_{t}^{i} \left( \mathrm{SQ}_{t+1},\mathrm{SQ}_{t} \right)=1-\sigma$.
  - They are susceptible in period $t$ and quarantine guarantees that they cannot be infected, so all other terms in that column are zero.
- People who are in state SNQ in period $t$ are the majority of the population – susceptible people not in quarantine.
  - They may be asked by the government to self-quarantine at a rate of $P_{t}^{i} \left( \mathrm{SQ}_{t+1},\mathrm{SNQ}_{t} \right)=w_{t}^{i}$. Usually, they self-quarantine either because they have been in close contact with a diagnosed patient, or because their demographic group is believed to have higher health risks once infected. We defined a parameter, ${sq}_{t}^{i}$, to represent the policy intensity about self-quarantine in Table 1. And the transition rate $w_{t}^{i}$ should satisfy $\delta w_{t}^{i}/\delta{sq}_{t}^{i}>0$ and $w_{t}^{i}=0$ when ${sq}_{t}^{i}=0$.
  - They may be infected at a rate of $P_{t}^{i} \left( \mathrm{IANQ}_{t+1},\mathrm{SNQ}_{t} \right)=\beta g_{t}^{i}\frac{IANQ_{t}}{SNQ_{t}+IANQ_{t}+R_{t}}$. Intuitively, as discussed in the Methods section of the paper, $\frac{IANQ_{t}}{SNQ_{t}+IANQ_{t}+R_{t}}$ is equivalent to $\frac{I}{N}$ in the standard SIR model, assuming quarantined and hospitalized people cannot meet with anyone. And $g_{t}^{i}$ is the social distancing parameter. A smaller value of $g_{t}^{i}$ corresponds to a lower transmission speed in the local community. This parameter can also represent the intensity of mask-wearing policies. Mathematically, the infection rate is the probability that an uninfected person meets an infected person multiplied by the probability that virus transmission takes place at the meeting, and by the number of meetings that happened in each time period.

Remark: Two reports from the World Health Organization stated that viral RNA is not equivalent to the ability to transmit the virus to another person [39, 52]. Therefore, we assume that a person can infect others as soon as she is infected, and do not differentiate the latent period from the incubation period. However, if future medical researches show that latent period is non-infectious, the users of this model should split each IA state into two states: a latent state and the existing asymptomatic state. People in these two states differ in their ability to infect others, domestic transmission ability, and travel behaviors.

- - The remaining people stay uninfected and out of self-quarantine. i.e. $P_{t}^{i} \left( \mathrm{SNQ}_{t+1},\mathrm{SNQ}_{t} \right)=1-w_{t}^{i}-\beta g_{t}^{i}\frac{IANQ_{t}}{SNQ_{t}+IANQ_{t}+R_{t}}$.
  - They are susceptible in period $t$, and the disease has an incubation period (with heterogeneous lengths of time), so all other terms in that column are zero.
- People who are in the state IAQ in period $t$ are people who were infected and in quarantine.
  - They may develop symptoms at a rate of $P_{t}^{i} \left( \mathrm{ISM}_{t+1},\mathrm{IAQ}_{t} \right)=\mu$,
  - Or recover without developing symptoms at a rate of $P_{t}^{i} \left( R_{t+1},\mathrm{IAQ}_{t} \right)=\gamma_{1}$,
  - Or stay infected in quarantine, i.e. $P_{t}^{i} \left( \mathrm{IAQ}_{t+1},\mathrm{IAQ}_{t} \right)=1-\mu-\gamma_{1}$.
  - They cannot be released from quarantine when infectious. We assume they cannot develop severe symptoms from asymptomatic states directly, so all other terms in that column are zero.
- People who are in the state IANQ in period $t$ are people who were infected but not in quarantine. We may treat these people as infectious patients who do not know they are infected.
  - They may develop mild symptoms at a rate of $P_{t}^{i} \left( \mathrm{ISM}_{t+1},\mathrm{IANQ}_{t} \right)=\mu$,
  - Or recover without developing symptoms at a rate of $P_{t}^{i} \left( R_{t+1},\mathrm{IANQ}_{t} \right)=\gamma_{1}$,
  - Or become quarantined at a rate of $h_{t}^{i}$, which is a function of $c_{t}^{i}$, $\theta_{t}^{i}$, $g_{t}^{i}$, and ${state}_{t}^{i}$, with $\delta h_{t}^{i}/\delta c_{t}^{i}>0$ and $h_{t}^{i}=0$ when $c_{t}^{i}=0$.
  - They may also stay in the current state, i.e. $P_{t}^{i} \left( \mathrm{IANQ}_{t+1},\mathrm{IANQ}_{t} \right)=1-\mu-\gamma_{1}-h_{t}^{i}$
  - We assume again that they cannot develop severe symptoms from asymptomatic states directly, so all other terms in that column are zero.
- People who are in the state ISM in period $t$ are people who have mild symptoms from the disease.
  - They may develop severe symptoms at a rate of $P_{t}^{i} \left( \mathrm{ISS}_{t+1},\mathrm{ISM}_{t} \right)=\tau$,
  - Or recover from mild symptoms at a rate of $P_{t}^{i} \left( R_{t+1},\mathrm{ISM}_{t} \right)=\gamma_{2}$.
  - They may continue to have mild symptoms, i.e. $P_{t}^{i} \left( \mathrm{ISM}_{t+1},\mathrm{ISM}_{t} \right)=1-\tau-\gamma_{2}$.
  - They cannot die without developing severe symptoms, so all other terms in that column are zero.
- People who are in the state ISS in period $t$ are people who have severe symptoms from the disease.
  - They may die at a rate of $P_{t}^{i} \left( D_{t+1},\mathrm{ISS}_{t} \right)=\delta$,
  - Or their condition may improve so that they only have mild symptoms at a rate of $P_{t}^{i} \left( \mathrm{ISM}_{t+1},\mathrm{ISS}_{t} \right)=\gamma_{3}$.
  - They may continue to have severe symptoms, i.e. $P_{t}^{i} \left( \mathrm{ISS}_{t+1},\mathrm{ISS}_{t} \right)=1-\delta-\gamma_{3}$.
  - They cannot recover without improving from ISS to ISM state, so all other terms in that column are zero.
- People who are in the state R in period $t$ are people who have recovered from the disease.
  - They may be reinfected at a rate of $P_{t}^{i} \left( \mathrm{IANQ}_{t+1},R_{t} \right)=\rho g_{t}^{i}\frac{IANQ_{t}}{SNQ_{t}+IANQ_{t}+R_{t}}$,
  - Or remain in the current state, i.e. $P_{t}^{i} \left( R_{t+1},R_{t} \right)=1-\rho g_{t}^{i}\frac{IANQ_{t}}{SNQ_{t}+IANQ_{t}+R_{t}}$.
  - We assume recovered people are similar to susceptible people except for the transmission rate, and they would not be asked to self-quarantine. So all other terms in that column are zero.
- People who are in state D in period $t$ are people who have died from the disease. Naturally, $P_{t}^{i} \left( D_{t+1},D_{t} \right)=1$.

**A1.2 Traveler’s characteristics and virus spread**

- The flow of travelers exiting area $i$ intending to enter area $j$ is characterized by

${travel}_{t}^{ij}=N_{t}^{ij}\times\left[ pr\left( \mathrm{SQ} \right),pr\left( \mathrm{SNQ} \right), pr\left( \mathrm{IAQ} \right), pr\left( \mathrm{IANQ} \right), pr\left( \mathrm{ISM} \right), pr\left( \mathrm{ISS} \right), pr\left( R \right),pr(D) \right]^{T}$ (8)

in which $N_{t}^{ij}$ is the number of travelers from area *i* to area *j*.

- Intuitively, ${travel}_{t}^{ij,\mathrm{SQ}}={travel}_{t}^{ij,I\mathrm{AQ}}={travel}_{t}^{ij,\mathrm{ISM}}={travel}_{t}^{ij,\mathrm{ISS}}={travel}_{t}^{ij,D}=0$, and $N_{t}^{ij}\leq N_{t}^{i}$.
- $\mathrm{IANQ}$ people, compared with $\mathrm{SNQ}$ and $R$ people, may have higher incentives to travel for less congested medical resources if the domestic condition in the destination area is better than the departure area. We define a crowd-out function $d^{ij}(.)$ that relates the fraction of IANQ people from area $i$ to area $j$, ${travel}_{t}^{ij,\mathrm{IANQ}}$, to the fractions of IANQ people in the two areas, i.e.

${travel}_{t}^{ij,\mathrm{IANQ}}=d^{ij}({state}_{t}^{i,\mathrm{IANQ}},{state}_{t}^{j,\mathrm{IANQ}})$ (9)

with ${travel}_{t}^{ij,\mathrm{IANQ}}\geq{state}_{t}^{i,\mathrm{IANQ}}$ if ${state}_{t}^{i,I\mathrm{ANQ}}>{state}_{t}^{j,\mathrm{IANQ}}$.

- It is more challenging for travelers to comply with social distancing due to the lack of space on trains and planes, so we denote the social distancing parameter by $g_{t}^{ij}$ with $g_{t}^{ij}>max\{g_{t}^{i},g_{t}^{j}\}$. Travel is a one-time-period activity without contact tracing or testing, so the contact tracing parameter for en route travelers is $c_{t}^{ij}=0$.

**A1.3 Travel policy upon arrival**

- When the travelers arrive at the destination area $j$, the government may choose to quarantine all inbound travelers for $q_{t}^{j}$ days.
- During the quarantine period, no infection among people takes place. The change in people’s compartments is only related to their own health status. Thus, the transition matrix for a one-day quarantine is time- and area-invariant, and is denoted by $\tilde{P}$.
- Before travelers are allowed to join the local population, the government may choose to test them for $r_{t}^{i}$ times. The effectiveness of the test, represented by the test’s type II error, is denoted by $\theta_{t}^{i}$.
- We denote the transition matrix corresponding to a one-time test by $\hat{P}_{t}^{i}$. In this matrix, $\hat{P}_{t}^{i}\left( \mathrm{ISQ}_{t+1},\mathrm{IAQ}_{t} \right)=\theta_{t}^{i}$ and $\hat{P}_{t}^{i}\left( \mathrm{IAQ}_{t+1},\mathrm{IAQ}_{t} \right)=1-\theta_{t}^{i}$. For other compartments A and B, $\hat{P}_{t}^{i}\left( A_{t+1},B_{t} \right)=1$ if and only if $A=B$, and zero otherwise.

**A1.4 A dynamic pandemic model evaluating reopening strategies**

We summarize the model by presenting the equation that relates ${state}_{t+1}^{i}$ to ${state}_{t}^{i}$ as

$${state}_{t+1}^{i}=P_{t}^{i}\left( {state}_{t}^{i}-\sum_{j\neq i} {travel}_{t}^{ij} \right)+\sum_{j\neq i} \sum_{x s.t. q_{t-x}^{i}=x} {{(\hat{P}_{t-x}^{i})}^{r_{t-x}^{i}}(\tilde{P})}^{x}P_{t-x}^{ij}{travel}_{t-x}^{ji}$$

which is equation (6).

The first part of the right hand side describes the evolvement of local people who did not exit the area. The second part describes the characteristics of all travelers who are out of their quarantine at time $t$.

To highlight the innovation of this model framework, Table A2 summarizes the policy interventions that are available to the government of area $i$.

**Table A2. Policy interventions considered in the model**

| **Policy type** | **Policy** | **Parameter** | **Range** |
| --- | --- | --- | --- |
| Internal | Social distancing | $g_{t}^{i}$ | $g_{t}^{i}\in(0,1]$ |
|  | Contact tracing and random testing | $c_{t}^{i}$ | $c_{t}^{i}\in[0,1]$ |
|  | Self-quarantine exit rate | $\sigma_{t}^{i}$ | $\sigma_{t}^{i}\in[0,1]$ |
|  | Self-quarantine intensity | ${sq}_{t}^{i}$ | ${sq}_{t}^{i}\in[0,1]$ |
| External | Number of travelers from area $j$ to area $i$ | $N_{t}^{ji}$ | $N_{t}^{ji}\in[0,N_{t}^{j}]$ |
|  | Number of travelers from area $i$ to area $j$ | $N_{t}^{ij}$ | $N_{t}^{ij}\in[0,N_{t}^{i}]$ |
|  | Number of quarantine days for travelers | $q_{t}^{i}$ | $q_{t}^{i}\geq0, q_{t}^{i}\in Z$ |
|  | Number of tests for travelers | $r_{t}^{j}$ | $r_{t}^{j}\geq0, r_{t}^{j}\in Z$ |
|  | Test effectiveness | $\theta_{t}^{i}$ | $\theta_{t}^{i}\in[0,1]$ |

**A2 Calibration**

The calibration intends to shed light on the current situation faced by many countries and regions amid COVID-19 outbreak. So we choose the specific parameter values to illustrate a simplified version of the real world.

**A2.1 Model setup**

To make clear inference from the model simulation, we consider three areas, i.e. low-risk, medium-risk, and high-risk.

Adopting the functional relationship between parameter values from the health literature and transition probabilities in the model in [24], we define the other nonzero entries in the local transition matrix $P$ in equation (5).

In the baseline model, we set the specific parameter values of these areas to mimic the condition of three countries in late July. We assess their domestic risk levels to index the areas by $L$ for the low risk area, $M$ for the medium risk area, and $H$ for the high risk area. The low risk area has experienced the first round of virus outbreak, and is considering reopening internally and externally. The medium risk area has contained one round of virus outbreak, and is at risk of a new round of outbreak due to a relaxation in social distancing. The high risk area failed to contain the virus in the early stage of the pandemic. It is experiencing a severe epidemic outbreak, and will have a larger fraction of recovered people than the other two areas. The model aims to analyze the epidemic and economic consequences of various reopening strategies of the low risk area, so we further simplify the calibration by assuming the absence of travelers between the medium and high risk countries, and symmetric travel capacities between each pair of areas, i.e. $N_{t}^{ij}=N_{t}^{ji}$. In the computer program, the flow of outbound traveler is defined as $N_{t}^{ji}=N_{t}^{ij}\times traveler ratio$, in which “traveler ratio” is a policy variable depending on the travel ban in the medium and high risk areas as well as the demand of travel to those areas. Interested readers can assign non-negative values to the “traveler ratio” when using the model.

We use the epidemiological parameter values from existing literature. Their definition, notation, value, and sources are summarized in Table A3.

**Table A3. Parameters of the model from the literature**

| **Parameter** | **Values** | **References** |
| --- | --- | --- |
| Average incubation period (days), $Incub$ | 5.2 | Li et al [38] |
| Average duration of illness (days) | 14 | World Health Organization [39] |
| Average length of stay in the ICU (days), $\mathrm{LoSICU}$ | 8.0 | Zhou et al [53] |
| Average duration of self-quarantine (days) | 14 | National Center for Immunization and Respiratory Diseases [54] |
| Infection fatality rate, $IFR$ | 0.006 | World Health Organization [40] |
| Basic reproduction number, $R_{0}$ | 2.79 | Australian Government Dept. of Health [55] |
| Fraction of patients with mild symptoms among all symptomatic patients, $\mathrm{Frac}\left( \mathrm{Mild} \right)$ | 0.8 | World Health Organization [56] |
| Fraction of asymptomatic patients among all infected people, $\mathrm{Frac}\left( \mathrm{IA} \right)$ | 0.4 | Sun et al [57], Oran et al [58] |

**A2.2 Specify the functions in the model**

We specify the functions in the model as follows:

- The probability that an IANQ person becomes quarantined is calculated by equating it with the average number of people that an IANQ person can infect in each time period.

$h\left( c_{t}^{i},\theta_{t}^{i},g_{t}^{i},{state}_{t}^{i} \right)=\sqrt{c_{t}^{i}\times\theta_{t}^{i}\times\mu\times\beta\times g_{t}^{i}\times\frac{{SNQ}_{t}^{i}}{{{SNQ}_{t}^{i}+IANQ}_{t}^{i}+R_{t}^{i}}}$ (10)

Proof: Denote $h\left( c_{t}^{i},g_{t}^{i},{state}_{t}^{i} \right)$ by $x$, and the average number of people that an IANQ person can infect by $y$. Consider a steady state that $x$ does not vary with state. The fraction of an IANQ person becoming quarantined equals the probability in which the person who infected him becomes IS times contact tracing probability times test accuracy. Mathematically, we have $x=\mu c_{t}^{i}\theta_{t}^{i}y$. On the other hand, the average number of people one can infect equals the number of people he can infect in each time period, multiplied by the expected duration of his stay in the IANQ period. Hence, we have

$$y=\left( \beta\times g_{t}^{i}\times\frac{{SNQ}_{t}^{i}}{{{SNQ}_{t}^{i}+IANQ}_{t}^{i}+R_{t}^{i}} \right)\times\sum_{n=1}^{\infty} x\left( 1-x \right)^{n-1}n (11)$$

Solving this pair of equations results in equation (10).

- The crowd-out effect among the travelers from area $i$ to area $j$ depends on the relative risk between the two areas. We determine the functional form and estimate the coefficient value as follows: The specific functional form of the function $d^{ij}$ is obtained through regression analysis using the data on daily new imported cases in China and the daily new confirmed cases in the US from March 29^th^ to April 15^th^, 2020 [59, 60]. The best-performing regression is $d_{t}^{ij}=0.342\times\sqrt{\mathrm{newcase}_{t-2}}+0.807\times\sqrt{\mathrm{newcase}_{t-3}}$, with an adjusted R-squared of 0.7554. The time lag in this serial correlation is likely to be caused by the delay in testing and reporting new cases from local areas to the central government in China. To restore the sampling bias of the traveler subpopulation from the overall population in the departure area, we adjust the equation as $d_{t}^{ij}=1.149\times\sqrt{\mathrm{newcase}_{t}}$. Given $P_{t}^{i} \left( \mathrm{IS}_{t+1},\mathrm{IANQ}_{t} \right)=\mu$, we conclude the amplification function presented in equation (12). When applying the model to a scenario without the crowd-out effect, one may use the identity function, i.e. $d^{ij}\left( {IANQ}_{t}^{i},{IANQ}_{t}^{j} \right)={state}_{t}^{i,\mathrm{IANQ}}$. Therefore, we assume that people who travel from a low risk area to a higher risk area are a representative sample of the population in the departure area.

$d^{ij}\left( {IANQ}_{t}^{i},{IANQ}_{t}^{j} \right)=\left\{ 1.149\times\begin{aligned} \sqrt{{{IANQ}_{t}^{i}}/{Incub}}, &{IANQ}_{t}^{i}>{IANQ}_{t}^{j} \\ {state}_{t}^{i,\mathrm{IANQ}}, &{IANQ}_{t}^{i}\leq{IANQ}_{t}^{j} \end{aligned} \right.$ (12)

- The probability that an SNQ person becomes quarantined is determined by government policy. For each new confirmed case, the government may choose how many people this person has interacted with should self-quarantine. The number of new confirmed cases on a given day can be estimated by $\frac{IANQ_{t}}{Incub}$. Therefore, one example of the functional form of $w_{t}^{i}$ could be $w_{t}^{i}=sq\times\frac{IANQ_{t}}{Incub}$. We use this functional form in the calibration, while the users of the model should use real world data to obtain a more realistic formula for $w_{t}^{i}$.

**A2.3 Initial states**

We determine the initial states of the three areas. In our calibration, the three areas are mainland China, Spain, and the U.S. The population distributions across the 8 states for each of the three areas are calculated from 4 numbers from actual data retrieved from [35-37] on July 29^th^, 2020: total death ($D$), total recovered ($R$), total cumulative confirmed ($I^{TOT}$), and the population size ($N$). We described the specific steps and their reasoning as follows and present the population distribution in the initial state of the model calibration in Table A4. Furthermore, we normalize the population sizes of the three areas to 1, i.e. $N^{L}=N^{M}=N^{H}=1$. In the computer program, the users of the model may follow the instructions we put in the code to revise the population sizes of the three areas based on their real-world scenario.

$pr \left( D \right)=D/N$.

$pr \left( R \right)=R/N$.

$pr\left( \mathrm{ISS} \right)+pr\left( \mathrm{ISM} \right)=\frac{I^{TOT}-R-D}{N}$. The share of currently infected symptomatic people is the share of total confirmed cases who are still alive and unrecovered.

$pr\left( \mathrm{ISS} \right)=\left( 1-\mathrm{Frac}\left( \mathrm{Mild} \right) \right)\times\left( pr\left( \mathrm{ISS} \right)+pr\left( \mathrm{ISM} \right) \right)$ and $pr\left( \mathrm{ISM} \right)=\mathrm{Frac}\left( \mathrm{Mild} \right)\times\left( pr\left( \mathrm{ISS} \right)+pr\left( \mathrm{ISM} \right) \right)$.

$pr\left( \mathrm{IANQ} \right)+pr\left( \mathrm{IAQ} \right)=\frac{1-\mathrm{Frac}\left( \mathrm{IA} \right)}{\mathrm{Frac}\left( \mathrm{IA} \right)}\times\left( pr\left( \mathrm{ISS} \right)+pr\left( \mathrm{ISM} \right) \right)$. This is by definition of $\mathrm{Frac}\left( \mathrm{IA} \right)$.

$pr\left( \mathrm{IAQ} \right)=h_{i}\times\frac{\left( pr\left( \mathrm{ISS} \right)+pr\left( \mathrm{ISM} \right) \right)}{\mathrm{aver}age duration of illness}\times\mathrm{ave}rage incubation period$. Each diagnosed patient corresponds to approximately $h_{i}=\sqrt{c_{i}\times\theta^{i}\times\mu\times\beta\times g_{i}}$ asymptomatic patients in quarantine. This number should be replaced by real-world estimates if the users of the model have it.

$pr\left( \mathrm{SQ} \right)$ is hard to identify from public information, so we set it to zero since it is very small. This number should also be replaced by real-world estimates when possible. Users of this model who have to assume an initial value for this number may consider the subsection on self-quarantine in the extension section of the paper as a reference to the impact of this assumption.

$pr\left( \mathrm{SNQ} \right)=1-pr \left( D \right)-pr\left( R \right)-pr\left( \mathrm{ISS} \right)-pr \left( \mathrm{ISM} \right)-pr \left( \mathrm{IAQ} \right)-pr \left( \mathrm{IANQ} \right)-pr\left( \mathrm{SQ} \right)$.

**Table A4. Initial states of the baseline model**

|  | **Parameter** | **Low risk area** | **Medium risk area** | **High risk area** |
| --- | --- | --- | --- | --- |
| Initial state  (${state}_{0}^{i}$) | $pr(SQ)$ | 0 | 0 | 0 |
|  | $pr(SNQ)$ | 0.9999213 | 0.9910 | 0.9620 |
|  | $pr(IAQ)$ | $3\times{10}^{-7}$ | 0.0001 | 0.0009 |
|  | $pr(IANQ)$ | $9\times{10}^{-6}$ | 0.0030 | 0.0192 |
|  | $pr(ISM)$ | $5\times{10}^{-6}$ | 0.0017 | 0.0107 |
|  | $pr(ISS)$ | $1\times{10}^{-6}$ | 0.0004 | 0.0027 |
|  | $pr(R)$ | $6\times{10}^{-5}$ | 0.0032 | 0.0040 |
|  | $pr(D)$ | $3\times{10}^{-6}$ | 0.0006 | 0.0005 |

**A2.4 Policy parameter values**

We assign policy parameter values in the baseline model. Table A5 reports the policy parameter values in the calibration. For the social distancing parameter $g$, we assign the values of 0.25, 0.7, and 1, respectively, mimicking different social distancing policies. When the model is used for actual policy evaluations, these parameters should be replaced by estimated values based real-life data. For the daily capacity of inbound travelers in percentage of the local population, we set the fully relaxed total capacity to 0.06%, which is close to the annual average in 2019 in the U.S [42]. For the baseline model, we set the total capacity to 0.01%, which is close to the March 2020 scenario in the U.S [42]. Please see Fig 2A for basic empirical patterns of the epidemic spreading in the three countries. The rate that SQ people rejoins the SNQ population, $\sigma$, equals the reciprocal of the average duration of self-quarantine. In the economic analysis, costs with positive values are presented in the main text of the paper. Users of the model should replace the economic measures with the actual cost in their scenario of interest.

**Table A5. Policy parameter values**

|  | **Parameter** | **Low risk area** | **Medium risk area** | **High risk area** |
| --- | --- | --- | --- | --- |
| Internal policies | $g_{t}^{i}$ | 0.25 | 0.7 | 1 |
|  | $c_{t}^{i}$ | 1 | 0.5 | 0.5 |
|  | $\sigma$ | 0.071 | - | - |
|  | ${sq}_{t}^{i}$ | 0 | - | - |
| External policies | $N_{t}^{ji}$ | $N_{t}^{ML}=N_{t}^{HL}=0.00005$, $N_{t}^{HM}=0$ | | |
|  | $N_{t}^{ij}$ | $N_{t}^{ij}=N_{t}^{ji}$ for $i,j\in\{L, M, H\}$ | | |
|  | $q_{t}^{i}$ | 7 | 0 | 0 |
|  | $r_{t}^{j}$ | 2 | 0 | 0 |
|  | $\theta_{t}^{i}$ | 0.8 | - | - |

**A2.5 Disease parameter values**

We use parameter values in Table A3 to calculate the values of the epidemiological parameters in the model. Table A6 shows the results.

**Table A6. Disease parameter values**

| **Parameter** | **Assigned value** | **Description** |
| --- | --- | --- |
| $\beta$ | 0.54 | Transmission rate |
| $\rho$ | 0 | Reinfection rate, i.e. the transmission rate for recovered people |
| $\gamma_{1}$ | 0.0634 | Recovery rate for asymptomatic patients |
| $\gamma_{2}$ | 0.0714 | Recovery rate for patients with mild symptoms |
| $\gamma_{3}$ | 0.12 | Improvement rate, the rate at which severe symptoms disappear |
| $\mu$ | 0.16 | Diagnosis rate |
| $\tau$ | 0.0158 | Severe symptom development rate |
| $\delta$ | 0.05 | Mortality rate of people with severe symptoms |

- $\beta=0.54$. The reproduction rate equals the daily transmission rate times the average incubation period.
- $\rho=0$. Reinfection cases have been reported but no existing studies have provided quantitative estimates for the reinfection rate. We assume $\rho=0$ in the baseline model, and discuss cases with $\rho>0$ in the extension section.
- $\gamma_{1}=0.0634$. For asymptomatic people, existing studies mentioned that they may recover without developing any symptoms, but information about their recovery rate is lacking [61]. Over the average duration of illness, the fraction of asymptomatic people who recovered without developing symptoms should equal the overall fraction of asymptomatic patients, i.e. $\left( {1-\gamma}_{1} \right)^{average duration of illness}=1-\mathrm{Frac}\left( \mathrm{IA} \right)$.
- $\gamma_{2}=0.0714$. The recovery rate for symptomatic patients equals to the reciprocal of the average duration of illness.
- $\gamma_{3}=0.12$. The expected value for severe patients to stay in the ISS state equals to the average length of stay in the ICU (denoted by LoSICU), including survivors and non-survivors, i.e.

$$\mathrm{LoSICU}=\sum_{n=1}^{\infty} \left( 1-\gamma_{3}-\delta\right)^{n-1}\left( \gamma_{3}+\delta\right)n$$

Solving the above equation, we obtain the formula for $\gamma_{3}$ as

$\gamma_{3}=\frac{1}{\mathrm{LoSICU}}-\delta$.

- $\mu=0.16$. Over the average incubation period, the fraction of patients who developed symptoms should equal the overall fraction of patients with symptoms, i.e. $\left( 1-\mu\right)^{Incub}$ $=\mathrm{Frac}\left( \mathrm{IA} \right)$.
- $\tau=0.0158$. The severe symptom development rate is the daily rate at which patients with mild symptoms develop severe symptoms. This rate, iterated over the average duration of illness, should generate the overall fraction of patients with severe symptoms, i.e. $\left( 1-\tau\right)^{average duration of illness}=Frac\left( \mathrm{Mild} \right)$.
- $\delta=0.05$. The mortality rate of patients with severe symptoms should align with the overall infection fatality rate, i.e. $IFR=\delta\times\left( 1-\mathrm{Frac}\left( \mathrm{Mild} \right) \right)\times\left( 1-\mathrm{Frac}\left( \mathrm{IA} \right) \right)$.

**A3 To use the model**

We encourage the readers of the article to make use of this model to enhance quantitative understanding of the reality, especially the ongoing COVID-19 pandemic. The Python code for our model is publicly available on GitHub at <https://github.com/zhongling1804/DynamicReopeningModel>.

Please note that the specific parameter values in the model are all based on public information as of August 3^rd^, 2020. When applying the model, please use the latest real-world data to estimate the parameter values, validate the model assumptions, and determine whether to adjust the functional forms.

**Code availability-** The code is available on GitHub for any reproduction and application.

**References**

52. Department of Communications, WHO Global. Transmission of SARS-CoV-2: implications for infection prevention precautions. 2020. Available from: Scientific Brief, WHO/2019-nCoV/Sci_Brief/Transmission_modes/2020.3.

53.Zhou F, Yu T, Du R, Fan G, Liu Y, Liu Z, et al. Clinical course and risk factors for mortality of adult inpatients with COVID-19 in Wuhan, China: a retrospective cohort study. The lancet. 2020 Mar 28;395(10229):1054-62.

54. National Center for Immunization and Respiratory Diseases (NCIRD), Division of Viral Diseases. 2021 Feb 1. Available from: https://www.cdc.gov/coronavirus/2019-ncov/travelers/after-travel-precautions.html

55. Australian Government Department of Health. Novel Coronavirus—Information for Clinicians. 2020. Available from: https://www.health.gov.au/sites/default/files/documents/2020/03/coronavirus-covid-19-information-for-clinicians.pdf.

56. World Health Organization. Coronavirus disease 2019 (COVID-19) Situation Report 46. 2020 March 6. Available from: https://www.who.int/docs/default-source/coronaviruse/situation-reports/20200306-sitrep-46-covid-19.pdf?sfvrsn=96b04adf_4.

57. Sun T, Weng D. Estimating the effects of asymptomatic and imported patients on COVID‐19 epidemic using mathematical modeling. Journal of medical virology. 2020 Apr 24.

58. Oran DP, Topol EJ. Prevalence of Asymptomatic SARS-CoV-2 Infection: A Narrative Review. Annals of Internal Medicine. 2020 Jun 3.

59. Wikipedia. 2019–20 coronavirus pandemic in the United States. 2020. In: Wikipedia, The Free Encyclopedia [Internet]. Available from: https://en.wikipedia.org/wiki/2020_coronavirus_pandemic_in_the_United_States.

60. Wikipedia. 2019–20 coronavirus pandemic cases in mainland China. 2020. In: Wikipedia, The Free Encyclopedia [Internet]. Available from: https://zh.wikipedia.org/wiki/2019%E5%86%A0%E7%8B%80%E7%97%85%E6%AF%92%E7%97%85%E4%B8%AD%E5%9C%8B%E5%A4%A7%E9%99%B8%E7%97%85%E4%BE%8B%E6%95%B8%E6%93%9A#%E5%A2%83%E5%A4%96%E8%BE%93%E5%85%A5%E7%97%85%E4%BE%8B.

61.Moghadas SM, Fitzpatrick MC, Sah P, Pandey A, Shoukat A, Singer BH, et al. The implications of silent transmission for the control of COVID-19 outbreaks. Proceedings of the National Academy of Sciences. 2020 Jul 28;117(30):17513-5.
